# Creating a nationwide cancer registration system to support AIDS-cancer match studies in Nigeria

**DOI:** 10.1186/1750-9378-7-S1-P3

**Published:** 2012-04-19

**Authors:** Clement Adebamowo, Elima Jedy-Agba, Emmanuel Oga, Peju Osinubi, Festus Igbinoba, Gloria Osubor, Theresa Otu, Henry Kumai, Michael Okobia, Prince Ejiroghene, Ahmed Mayun, James Abdulazeez, O Erinomo, Adebayo Ojo, Cornelius Uka, Gloria Oyeoka

**Affiliations:** 1 Nigerian National Cancer Registry System

## Background

Cancer registration started in Nigeria in 1962 but after a very promising start, the momentum was lost [1,2]. Since 2009, Nigerian Health Ministry, IARC and IPRI, have given training, troubleshooting and mentoring.

## Material and methods

20 cancer registries were trained and 5 have met criteria for population based cancer registries. Data for 2009 are presented in this report.

## Results

The commonest cancer at all sites is Prostate in men and Breast in women. There was a gradient in the incidence that paralleled the socio-economic development of the regions of the country. The ASR for breast cancer ranged from 101.1 in Abuja to 7.5 in less cosmopolitan areas. For Prostate the ASR ranged from 73 in Abuja to 1.7. The other common cancers were Kaposi Sarcoma and Colo-Rectal in men, and cervix in women. Additional data collection and analysis is ongoing.

## Conclusions

This study showed that breast and cervical cancer are the commonest in women while prostate is the commonest cancer in men.
